# Proximity to transplant center and outcome among liver transplant patients

**DOI:** 10.1111/ajt.15004

**Published:** 2018-08-03

**Authors:** Gwilym J. Webb, James Hodson, Abhishek Chauhan, John O’Grady, James M. Neuberger, Gideon M. Hirschfield, James W. Ferguson

**Affiliations:** ^1^ Liver Medicine University Hospitals Birmingham Birmingham UK; ^2^ National Institute for Health Research Birmingham Liver Biomedical Research Unit University of Birmingham Birmingham UK; ^3^ Statistics, Institute of Translational Medicine University Hospitals Birmingham Birmingham UK; ^4^ Institute of Liver Studies King's College Hospital NHS Foundation Trust London UK

**Keywords:** business / management, clinical research / practice, disparities, health services and outcomes research, informatics, liver disease, liver transplantation / hepatology, organ transplantation in general, patient characteristics, patient referral

## Abstract

In the United States, distance from liver transplant center correlates with worsened outcomes; the effects of geography elsewhere are unassessed. We performed a national registry analysis of United Kingdom listings for liver transplantation (1995‐2014) and assessed whether travel time to transplant center correlates with outcome. There were 11 188 listings assessed (8490 transplanted), with a median travel time to center of 60 minutes (range 36‐86). Of the national population, 3.38 × 10^7^ (55.1%) reside ≥60 minutes from a center, and 7.65 × 10^6 ^(12.5%) >119 minutes. After competing risk analysis, increasing travel time was associated with an increased risk of death after listing (subdistribution hazard ratios relative to <60 minutes of 1.33 for 60‐119 and 1.27 for >119 minutes; *P* < 0.001) and reduced likelihood of transplantation or recovery (0.94 and 0.86; *P* < 0.001). Among those transplanted, travel time was not associated with retransplant‐free survival (*P* = 0.532). We used our model to examine optimal placement of a new center and identify a single site with a total travel time reduction of ≈10%. Our findings of disparities in accessibility of liver transplantation showed worse outcomes following listing in those distant from their transplant center, and our description of a method to model a new center complement existing data and support similar analyses of other networks.

## Abstract 2

This analysis demonstrates worse outcomes for UK patients listed for liver transplantation who live further from a transplant center, describes the current geography of the UK transplant population, and models the optimal position for an additional center to reduce access inequality. Hanto and Ladin's editorial is on page 13.

**Abbreviations**
ASMR: adjusted standardized mortality ratio
IQR: interquartile range
UK NHS: United Kingdom National Health Service

## 1 INTRODUCTION

Liver transplantation saves lives in liver failure and hepatocellular carcinoma and, in the context of an ever‐increasing demand for transplantation, promoting equality of access has become a focus.^1^, ^2^, ^3^, ^4^ Recent reports from the United States have described worsened outcomes for liver transplantation for those living at greater distances or travel times from transplant centers; the effect is seen prior to listing, from listing, and from the point of transplantation.^5^, ^6^ Such worsened outcomes are also reported for the transplantation of other solid organs^7^ and for treatment of cancer for those living at greater distances from specialist cancer care centers.^8^ Conversely, improved outcomes are reported for centralization of services for certain specialist procedures (eg, surgery for pancreatic cancer in the United Kingdom, and for liver transplantation in the United States).^9^, ^10^

The United Kingdom National Health Service (UK NHS) has a well‐established liver transplant program, and represents the only option for liver transplantation within the United Kingdom. Since 1992, the NHS provision of care has been split between 7 centers. Recent analyses of the provision of national liver transplant services have emphasized reducing disparity and considering the building of new transplant center(s) to do so.^11^, ^12^, ^13^, ^14^ In addition to potential effects on patient outcome, it is reported that proximity to treating transplant center is identified as a factor important to many liver transplant patients.^15^ To date, however, the roles of distance and travel time in UK liver transplantation have not been formally assessed.

In this analysis, we sought to describe the geographic distribution of UK patients using liver transplantation services, assess whether current variations in travel time are associated with differences in outcome, and to assess where a potential new liver transplant center might be best placed to minimize travel time.

## 2 MATERIALS AND METHODS

**Figure 1:**
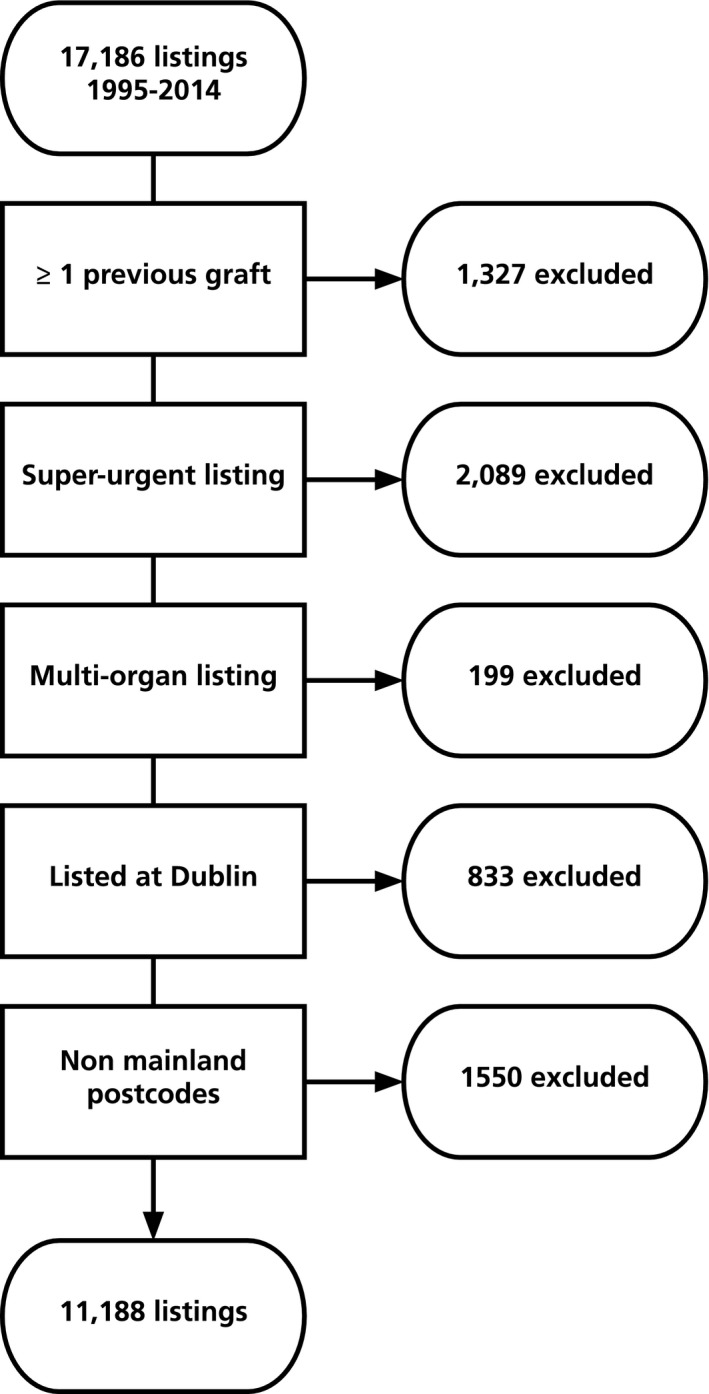
Flowchart of exclusions. Adult patients (17 186) were listed for liver transplantation from 1995 to 2014 inclusive. After application of the exclusion criteria, an analysis cohort of 11 188 was generated

The NHS Blood and Transplant national registry was queried for all patients ≥18 years old listed for liver transplantation in the United Kingdom from 1995 to 2014 inclusive; approval for the study was given by the UK Transplant Registry. Patients listed for repeat transplants, those listed on a “super‐urgent” basis, those listed for simultaneous multi‐organ transplantation, and those listed from postal codes (postcodes) outside England, Scotland, or Wales were excluded (Figure 1). Those resident in Northern Ireland were excluded because of the incomplete availability of Census data, incomplete availability of data on population liver‐related mortality, and the distorting effects of air travel. Data sources are summarized in Table S1.

To preserve anonymity, only the first portion of each patient’s postcode was available describing the “postcode district.” The longitude and latitude for the centroid of each of 2736 postcode districts (Figure S1) and the precise location of each renal or liver transplant center was then entered into the Google Maps API (Alphabet, Palo Alto, CA). The shortest driving distance and travel time, unadjusted for traffic conditions, were then calculated from each postcode district to each transplant center. Centers with a preexisting renal transplant center were chosen as a proxy for the presence of sufficient infrastructure to support a new liver transplant center. Travel times were divided into 3 groups: <60 minutes, 60‐119 minutes, and >119 minutes. Population estimates for postcode districts were obtained from the 2011 UK National Census; adjusted standardized mortality ratio (ASMR) estimates for liver disease were obtained for 2011 from national agencies. Geographic boundaries of the organizational subunits for which ASMR was available were overlaid with postcode districts and each postcode district was assigned the ASMR for the healthcare administrative area with the most shared area. Mapping was performed using Quantum GIS v2.18.7 (https://qgis.osgeo.org).

For the assessment of outcome from listing, death on the waiting list and de‐listing for worsening condition were treated as outcomes, with patients censored if they received a transplant or were de‐listed because of an improvement in their condition. For the assessment of outcome from transplantation, retransplant‐free survival was assessed. Here, the outcomes of interest were mortality and the receipt of a second transplant, with patients censored at the end of follow‐up. Assessments were performed with both travel time and travel distance as both categorical and continuous variables. Primary liver diagnoses were categorized into the following categories: primary biliary cholangitis, autoimmune hepatitis, hepatitis B virus, primary sclerosing cholangitis, alcohol, hepatitis C virus, nonalcoholic fatty liver disease, or other. The presence or absence of hepatocellular carcinoma was coded separately from primary diagnosis. Those patients with missing data in 1 or more categories were excluded from the univariable analyses in question and entirely from multivariable analyses.

Univariable analyses were used to compare differences between the 3 travel time categories: the nptrend test in Stata for continuous variables,^16^ the Mann‐Whitney *U* test to compare travel time between 2 categorical variables, and the Kruskal‐Wallis test to compare 3 or more categorical variables. To assess for outlying contributors of listings for liver transplantation, the relative contribution of different postcode areas—each including multiple postcode districts—to listings for liver transplantation, rates, and confidence intervals were calculated to generate a funnel plot according to the method of Spiegelhalter.^17^

To assess outcome from the point of listing for transplantation, competing‐risks regression models according to the method of Fine and Gray were constructed.^18^ Analyses were constructed both with death as the primary outcome and transplantation or recovery as a competing risk, and also with attaining transplantation or recovery as a primary outcome and death or all other outcomes as a competing risk. To assess outcome following liver transplantation, a Cox proportional hazards model was constructed with the incidence of either death (of any cause) or retransplantation considered a failure.

In both competing risk and Cox models, variables other than travel time were selected for inclusion in the final model with a backwards stepwise approach with a cut‐off of *P* < .1. Visual inspection was used to ensure no crossover of Kaplan‐Meier survival plots for each categorical value. For Fine‐Gray competing risk models, cumulative sums of residuals were used to confirm the appropriateness of the model constructed for each variable; for Cox models, Martingale and Cox‐Snell residuals were calculated.^19^ For skewed values with poor fit, logarithmic transforms were used (this was required in 2 instances: intensive treatment unit stay duration and international normalized ratio). A value of *P* < .05 was assumed to be representative of statistical significance. Statistical analyses were conducted using StataMP v15.0 (StataCorp, College Station, TX) using the University of Birmingham’s BlueBEAR High Performance Computing Cluster.

## 3 RESULTS

Adult listings (17 186) for liver transplantation were identified. After exclusions, 11 188 were included in subsequent analysis (Figure 1). Data on distance and travel time were available for 11 184 of 11 188 transplants (>99.9%), with the remainder not having a valid recorded postal code. The median distance to the nearest center was 67.3 km (interquartile range [IQR] 23.6‐107.6); median travel time was 60 minutes (IQR 36‐86).

**Figure 2:**
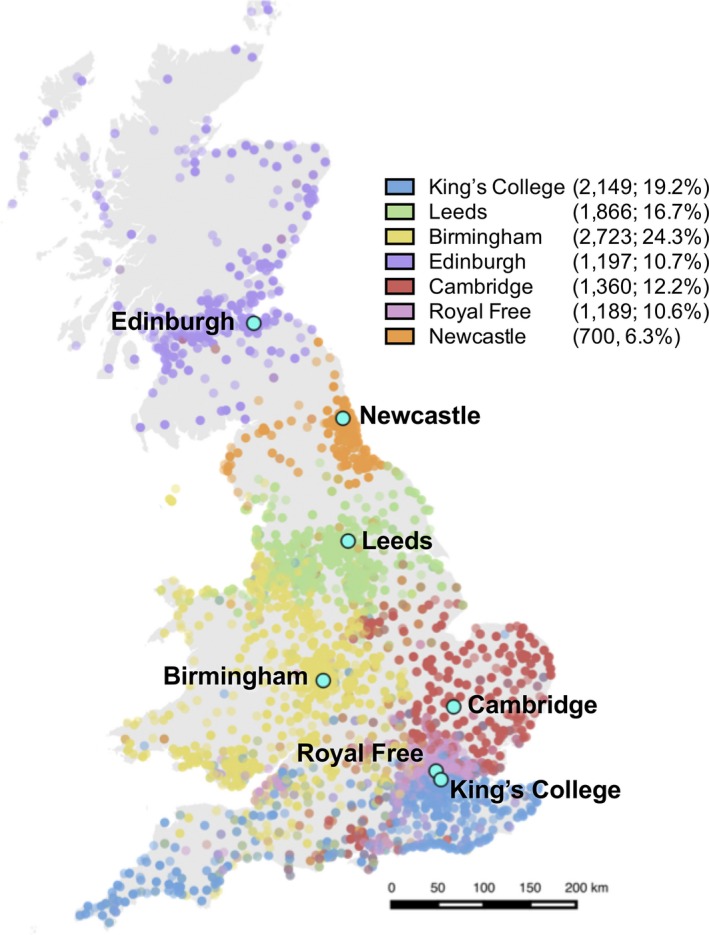
Geographic distribution of listings for liver transplantation. Geographic distribution of patients listed for liver transplantation in England, Scotland, and Wales 1995‐2014 inclusive after the exclusions detailed in Figure 1 [Color figure can be viewed at wileyonlinelibrary.com]

Travel time to nearest transplant center was closely correlated with distance from nearest transplant center (*r* ^2^ = 0.913; *P* < .001) (Figure S2). For the remainder of the analysis, travel time was used as the primary comparator. The majority of patients attended their nearest liver transplant center (8505 of 11 184; 76.0%) (Figure 2). Those who did not attend their nearest center were primarily resident in areas of near equidistance between centers, or in the northwest or southwest of England (Figure S3).

**Figure 3:**
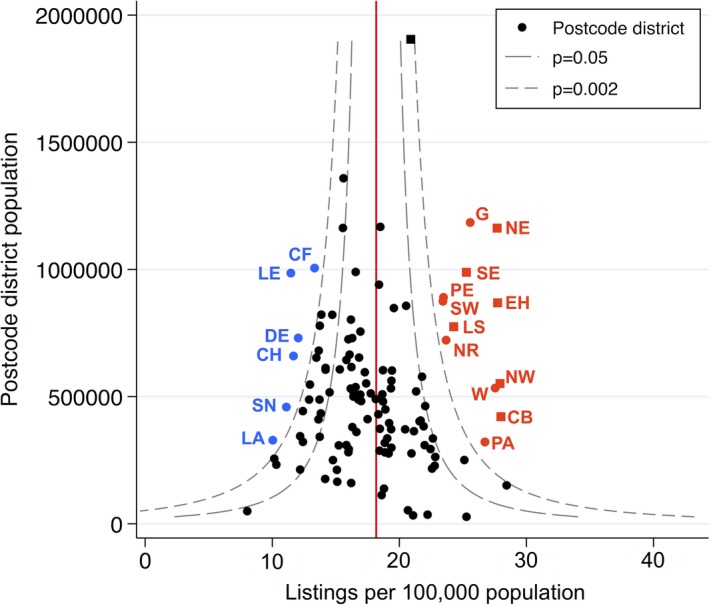
Listings for liver transplantation by postcode area. The number of listings for liver transplantation 1995‐2014 for each postcode area (n = 2736) was aggregated from the constituent postcode area and plotted against the 2011 National Census population. Confidence intervals at *P = .*05 and .002 were then generated to assess for those postcode areas contributing either more or fewer than expected listings. Those areas contributing fewer listings than expected at *P* < .002 are marked in blue; those contributing more at *P* < .002 are marked in red; text labels represent postcode areas. Postcode areas containing a liver transplant center (7) are plotted with a square rather than a circular marker [Color figure can be viewed at wileyonlinelibrary.com]

Several postcode areas supplied a disproportionate number of total listings as a proportion of the resident population as at the 2011 National Census population (Figure 3) at a confidence level of 3 standard deviations (*P* < .002). Among these were 6 out of 7 postcode areas containing one of the liver transplant centers; the seventh transplant center—Birmingham—was located in a postcode area that contributed an excess of listings at *P* < .05 but not *P* < .002. Among postcode areas over the study period, there was a median of 22.1 listings/100 000 population (IQR 18.8‐26.7).

### 3.1 There is an uneven distribution of access to liver transplant centers across the United Kingdom

**Figure 4:**
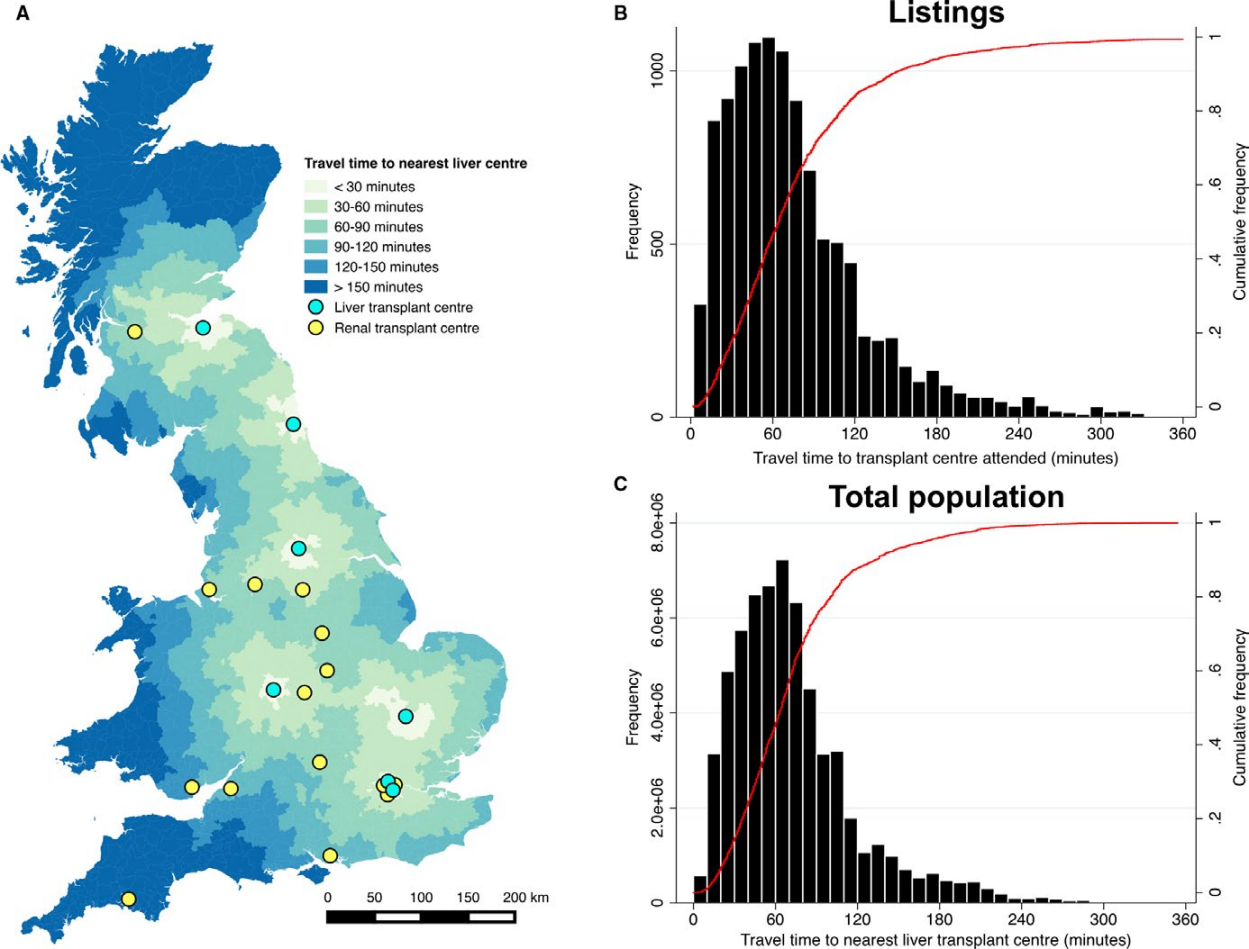
Travel time to nearest liver transplant center. A, Map of Great Britain showing the locations of current liver transplant centers (cyan circles), renal transplant centers without liver transplant capability (yellow circles), and calculated travel time to the nearest liver transplant center from each postcode district in 30‐minute intervals. B, Frequency distribution of travel time from postcode district of residence of patients listed for transplantation to the liver transplant center attended (n = 11 188). Red line denotes cumulative frequency. C, Frequency distribution of travel time from postcode district of residence of total resident population to the nearest liver transplant center (n = 6.13 × 10^7^). Red line denotes cumulative frequency [Color figure can be viewed at wileyonlinelibrary.com]

When calculated across the United Kingdom, marked variation in travel time to the nearest center was evident (Figure 4A). Of those 11 184 listed for transplantation, 5620 (50.3%) were resident ≥60 minutes from a liver transplant center, 1262 (11.3%) were >119 minutes away, and 494 (4.4%) were ≥180 minutes away (Figure 4B). With respect to the general population of Great Britain, as measured in the 2011 National Census (n = 6.13 × 10^7^), approximately 3.38 × 10^7^ (55.1%) people were resident ≥60 minutes from a liver transplant center, 7.65 × 10^6 ^(12.5%) were >119 minutes away, and 2.49 × 10^6 ^(4.0%) were ≥180 minutes away (Figure 4C).

To assess whether there was a correlation between mortality from liver disease with travel time to nearest liver transplant center, we plotted ASMR for each postcode district against travel time. This revealed a negative correlation −3.51 ASMR points per 100 minutes of travel time (95% confidence interval [CI] to −2.97 to −4.04; *P* < .001; *r* ^2^ = 0.061; Figures S4 and S5).

To simultaneously consider both population density and travel time to the nearest transplant center, we calculated the product of the resident population of each postcode district and the travel time to the nearest transplant center (Figure S6). The largest values of “person minutes” generated related to southwest England, south Wales, and around urban centers in northern England. We then repeated this procedure but adjusted values according to the ASMR for that postcode district normalized to the average ASMR nationwide (Figure S7). After this adjustment, the largest values of “person minutes” were evident in the northwest of England.

### 3.2 Travel time is significantly correlated with worsened outcome from the point of listing for liver transplantation and a lower likelihood of receiving a liver transplant

To assess outcome after listing of transplant patients, we first divided patients into 3 groups: <60 minutes, 60‐119 minutes, and >119 minutes travel time to the center used. Of the 11 184 patients listed, 8865 were transplanted or removed because of an improvement in their condition, 2228 had died or were removed because of deterioration in their condition, and 95 were still active on the transplant list. Median follow‐up from listing was 75 days (IQR 26‐179 days). Of those who died or were delisted for worsening, median time to event was 96 days (IQR 34‐227 days).

The results of univariable analyses comparing average travel times across a range of factors are presented in Tables 1 and 2. Within the cohort of listed patients, travel times were found to differ significantly by ethnicity (*P* < .001), being longest in white patients; by blood group (*P* < .001), being shortest for group B potential recipients; and by liver disease, being shortest in viral hepatitis. Serum bilirubin was also found to increase significantly with travel time (*P* = .012), and travel time increased over the study period (*P* < .001), with a significant difference in travel time between transplant units (*P* < .001).

**Table 1:** Study population characteristics

Variable	Category	Listed patients			Transplanted patients		
		n	Median (IQR) travel time (min)	*P*	n	Median (IQR) travel time (min)	*P*
Age (years)	≤40 y	1733	64 (40‐100)	.151	1296	63 (40‐100)	.156
	>40 ≤ 55 y	4725	64 (38‐97)		3593	63 (37‐97)	
	>55 y	4722	65 (39‐101)		3520	65 (38‐101)	
Sex	Male	7011	64 (38‐100)	.289	5298	63 (37‐100)	.333
	Female	4168	65 (39‐98)		3110	65 (39‐99)	
BMI (kg/m^2^)	<25	4517	65 (39‐100)	.795	3349	65 (39‐100)	.838
	≥25 < 30	3539	64 (37‐102)		2760	63 (37‐102)	
	≥30	2493	65 (40‐100)		1818	65 (40‐100)	
Recipient ethnicity	White	9810	68 (41‐103)	**<.001**	7417	67 (41‐103)	**<.001**
	Asian	961	45 (26‐65)		711	45 (26‐65)	
	Black	271	35 (19‐57)		194	34 (18‐55)	
	Other/mixed	132	35 (15‐59)		86	32 (12‐56)	
Serum bilirubin (µmol/L)	≤30	2347	64 (37‐100)	**.012**	1668	65 (37‐100)	**.043**
	>30 ≤ 60	2025	66 (41‐100)		1501	65 (40‐98)	
	>60	2834	66 (42‐100)		1993	65 (42‐101)	
Serum sodium (mmol/L)	≤135	2741	67 (41‐100)	.407	1885	66 (40‐100)	.707
	>135 ≤ 140	3285	64 (40‐98)		2400	64 (40‐98)	
	>140	1163	65 (40‐105)		864	65 (40‐105)	
INR	≤1.2	2663	66 (40‐105)	.112	1910	67 (40‐105)	.077
	>1.2 ≤ 1.6	2991	65 (40‐98)		2167	65 (40‐98)	
	>1.6	1512	64 (41‐95)		1056	64 (40‐95)	
Serum creatinine (µmol/L)	≤60	1295	63 (39‐101)	.138	945	63 (38‐100)	.261
	>60 ≤ 90	3418	65 (40‐100)		2589	65 (40‐100)	
	>90	2475	66 (41‐100)		1615	66 (40‐100)	
Blood group	O	5086	65 (39‐100)	**<.001**	3567	64 (38‐100)	**.001**
	A	4321	66 (40‐102)		3520	65 (39‐100)	
	B	1325	55 (34‐90)		944	56 (34‐93)	
	AB	448	63 (36‐92)		378	65 (36‐94)	
Diabetes mellitus	No	9937	64 (38‐99)	.203	7531	64 (38‐100)	.425
	Yes	1243	65 (40‐101)		878	65 (39‐100)	
Hemodialysis	No	11056	64 (39‐100)	.414	8350	64 (38‐100)	.885
	Yes	124	60 (37‐89)		59	63 (38‐98)	
Liver disease	PBC	1376	66 (42‐102)	**<.001**	1162	66 (41‐102)	**<.001**
	AIH	494	70 (42‐102)		386	68 (42‐100)	
	HBV	436	47 (25‐84)		352	47 (24.5‐84)	
	PSC	1027	65 (41‐101)		854	66 (41‐102)	
	Other	1749	65 (40‐105)		1100	65 (38.5‐104)	
	Alcohol	2920	65 (38‐98)		2172	63 (38‐99)	
	HCV	1868	61 (35‐94)		1454	60 (35‐95)	
	NAFLD	428	66 (45‐99)		288	67 (46‐98)	
HCC	No	9797	65 (39‐99)	.551	7324	64 (38‐99)	.524
	Yes	1383	63 (36‐104)		1085	63 (36‐100)	
Transplant unit	King’s College	2148	63 (37‐108)	**<.001**	1585	62 (36‐108)	**<.001**
	Leeds	1866	61 (39‐78)		1312	58 (39‐78)	
	Birmingham	2723	93 (47‐123)		2095	93 (49‐123)	
	Edinburgh	1196	67 (50‐96)		920	66 (49‐94)	
	Cambridge	1359	75 (52‐96)		1074	75 (52‐95)	
	Royal Free	1188	44 (31‐62)		894	44 (31‐61)	
	Newcastle	700	32 (20‐54)		529	32 (20‐55)	
Listing year	1995‐1999	2053	59 (33‐99)	**<.001**	1708	60 (34‐99)	**.003**
	2000‐2004	2396	65 (39‐98)		1963	65 (39‐99)	
	2005‐2009	2833	65 (40‐97)		2000	64 (39‐97)	
	2010‐2014	3898	65 (40‐101)		2738	65 (40‐101)	

AIH, autoimmune hepatitis; BMI, body mass index; INR, international normalized ratio; HBV, hepatitis B virus; HCC, hepatocellular carcinoma; HCV, hepatitis C virus; IQR, interquartile range; NAFLD, nonalcoholic fatty liver disease; PBC, primary biliary cholangitis; PSC, primary sclerosing cholangitis.

Values represent the median travel time for that category. The Mann‐Whitney test was used to compare travel times between 2 categorical variables; the Kruskal‐Wallis test was used to compare 3 or more categorical values; the nptrend test was used to test for trend across numerical values between tertiles. Blood results are from the point of listing. Bold *P* values are significant at *P* < .05.

**Table 2:** Additional transplant‐related patient characteristics

Variable	Category	n	Median (IQR) travel time (min)	*P*
Donor BMI (kg/m^2^)	<25	3843	63 (37‐96)	**.004**
	≥25 < 30	2411	64 (38‐103)	
	≥30	1183	66 (40‐102)	
Donor age (y)	≤40	2978	63 (38‐98)	.122
	>40 ≤ 55	2941	63 (38‐97)	
	>55	2490	65 (39‐102)	
Cold ischemic time (min)	≤500	2792	65 (40‐101)	**.004**
	>500 ≤ 750	4137	64 (38‐98)	
	>750	1480	61 (34‐97)	
Days in ITU (d)	1	2402	62 (38‐89)	**<.001**
	2 or 3	3543	65 (38‐103)	
	>3	2175	65 (38‐109)	
Transplant weekday	Sunday	1046	65 (37‐107)	.726
	Monday	1123	62 (36‐100)	
	Tuesday	1315	65 (38‐101)	
	Wednesday	1377	63 (39‐96)	
	Thursday	1233	64 (38‐100)	
	Friday	1199	63 (39‐95)	
	Saturday	1116	65 (41‐98)	
CMV status	D−R−	1447	68 (43‐105)	**<.001**
	D−R+	2263	59 (35‐93)	
	D+ R+	2280	61 (35‐100)	
	D+ R−	1399	69 (44‐103)	
Donor sex	Male	4488	66 (39‐102)	**<.001**
	Female	3921	62 (37‐95)	
Donor ethnicity	White	6914	65 (39‐100)	**.022**
	Asian	138	50 (31‐85)	
	Black	85	60 (37‐90)	
	Other/mixed	70	59 (35‐84)	
Graft type	DBD	7467	64 (38‐100)	.645
	DCD	941	65 (39‐100)	

BMI, body mass index; CMV, cytomegalovirus; D, donor; DBD, donation after brain death; DCD, donation after cardiac death; ITU, intensive treatment unit; IQR, interquartile range; R, recipient.

Values either represent the median of the characteristic concerned or the median travel time for that category. The Mann‐Whitney test was used to compare travel times between 2 categorical variables; the Kruskal‐Wallis test was used to compare 3 or more categorical values; the nptrend test was used to test for trend across numerical values between tertiles. Bold *P* values are significant at *P* < .05.

**Figure 5:**
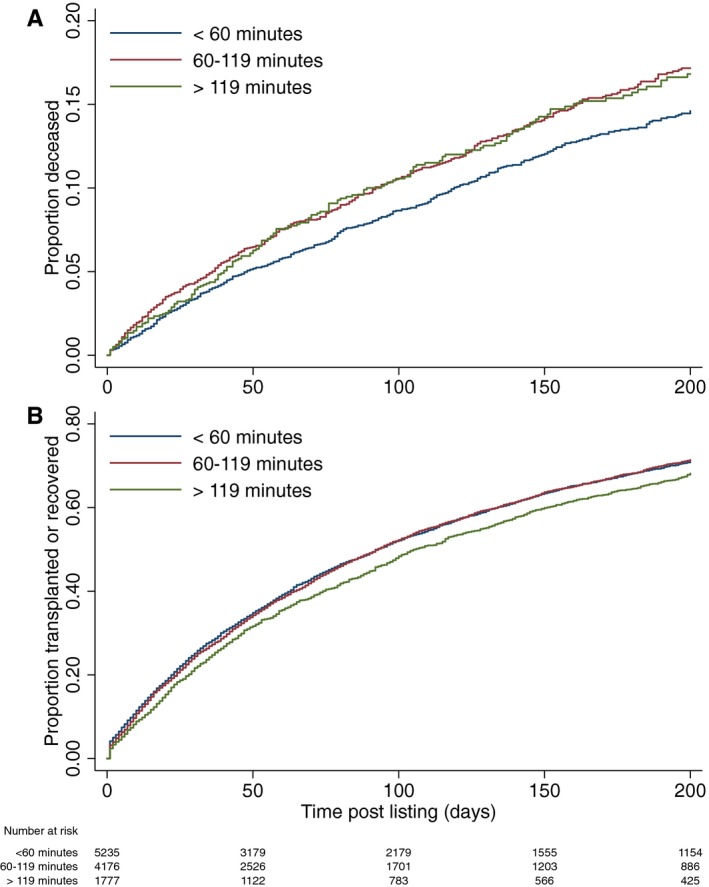
Outcome following listing for liver transplantation. A, Proportion of patients who had died following listing for liver transplantation categorized by travel time to listing transplant center (n = 11 188; *P = *.003 by log‐rank test). B, Proportion of patients who received transplantation or were removed from the waiting list because of an improvement in their clinical condition following listing for liver transplantation categorized by travel time to listing transplant center (n = 11 188; *P = *.005. by log‐rank test) [Color figure can be viewed at wileyonlinelibrary.com]

An unadjusted analysis of postlisting survival demonstrated significant differences between travel‐time categories (Figure 5A; *P = .*003 by log‐rank test), with a hazard ratio of 1.21 (95% CI: 1.03‐1.41, *P = .*020) for the >119 minutes vs <60 minutes groups. However, this did not account for the rates of transplantation or recovery, which were also found to differ significantly across the groups, being lowest in the >119 minutes group (HR: 0.91; 95% CI: 0.85‐0.96, *P = .*002, Figure 5B). As such, competing risks analyses were performed, to consider both of these outcomes simultaneously. These models also accounted for other confounding factors, in order to account for the baseline differences observed between the 3 travel time groups.

When considering death as the primary outcome (Table 3), the model found survival to be shorter in those with longer travel times (*P* < .001), with subdistribution hazard ratios (sHRs) of 1.33 (95% CI: 1.12‐1.57, *P = .*001) for the 60‐119‐minute group, and 1.27 (1.01‐1.59, *P = .*037) for the >119‐minute group, relative to the <60‐minute group. When the competing risks model was reanalyzed with travel time as a continuous variable, there was a significant correlation with a greater risk of mortality with increasing travel time to transplanting center: sHR 1.09 (per 60 minutes, 95% CI: 1.02‐1.17, *P = .*013).

**Table 3:** Risk of death from the point of listing for liver transplantation

Variable	sHR (95% CI)	*P*
Travel time tertiles	**—**	**<.001**
<60 min	1.00	—
60‐119 min	1.33 (1.12‐1.57)	**.001**
>119 min	1.27 (1.01‐1.59)	**.037**
Age (/10 y)	1.24 (1.15‐1.34)	**<.001**
Serum bilirubin (/100 µmol/L)	1.23 (1.17‐1.30)	**<.001**
Serum sodium (/10 mmol/L)	0.55 (0.48‐0.64)	**<.001**
Serum creatinine (/100 µmol/L)	1.25 (1.15‐1.36)	**<.001**
INR (per 2‐fold increase)	2.09 (1.60‐2.74)	**<.001**
Transplant center	—	**.002**
King’s College	1.00	**—**
Leeds	0.95 (0.74‐1.23)	.705
Birmingham	0.89 (0.71‐1.12)	.328
Edinburgh	0.90 (0.69‐1.17)	.432
Cambridge	0.57 (0.42‐0.77)	**<.001**
Royal Free	0.91 (0.69‐1.22)	.532
Newcastle	0.56 (0.37‐0.84)	**.006**
HCC	—	**—**
No	1.00	**‐**
Yes	0.35 (0.25‐0.48)	**<.001**
Blood group	—	**<.001**
O	1.00	**—**
A	0.63 (0.53‐0.75)	**<.001**
B	1.20 (0.96‐1.49)	.102
AB	0.56 (0.36‐0.89)	**.015**
Primary liver disease	—	**<.001**
PBC	1.00	**—**
AIH	0.95 (0.62‐1.45)	.803
HBV	0.84 (0.48‐1.47)	.543
PSC	0.62 (0.43‐0.90)	**.012**
Other	1.69 (1.27‐2.25)	**<.001**
Alcohol	0.94 (0.71‐1.23)	.642
HCV	1.03 (0.76‐1.41)	.835
NAFLD	1.22 (0.85‐1.74)	.275
Ethnic group	—	.150
White	1.00	**—**
Asian	0.71 (0.51‐0.98)	**.038**
Black	0.99 (0.59‐1.66)	.965
Other/mixed	1.33 (0.64‐2.77)	.447

AIH, autoimmune hepatitis; BMI, body mass index; CI, confidence interval; HBV, hepatitis B virus; HCC, hepatocellular carcinoma; HCV, hepatitis C virus; INR, international normalized ratio; NAFLD, nonalcoholic fatty liver disease; PBC, primary biliary cholangitis; PSC, primary sclerosing cholangitis.

Variables presented represent those from Table 1 retained in the competing risks model after backwards stepwise selection: covariables excluded because of the absence of a significant effect on outcome were: sex, BMI, INR, diabetes mellitus, hemodialysis, and listing year. Subdistribution hazard ratios (sHR) are presented along with upper and lower 95% CI. Only listings with complete data are included (n = 6744). The sHR calculated for INR and days in intensive treatment unit relates to an increase of 1 in the log_2_‐transformed variable (eg, a 2‐fold increase in INR). For the other continuous factors, sHRs are for increases of the stated number of units. Blood results are from the point of listing. Bold *P *values are significant at *P* < .05.

A similar competing risks model to the one described above was constructed but with attaining transplantation or recovering to the point of no longer needing a transplant as the outcome of interest, with death or delisting for a decline in condition as a competing risk. Here there was a reduced likelihood of receiving a transplant associated with longer travel time, with sHRs of 0.94 (0.88‐0.99, *P* = .039) for the 60‐119‐minute group and 0.86 (95% CI: 0.79‐0.93, *P* < .001) for the >119‐minute group, relative to the <60‐minute group (Table S2). When the model was reanalyzed with travel time as a continuous variable, there was a significant correlation with a reduced likelihood of receiving a liver transplant and increasing travel time to transplanting center: sHR 0.95 (0.93‐0.98; per 60‐minute travel time), *P* = .001.

### 3.3 Travel time is not significantly correlated with outcome following liver transplantation

We next looked for differences in outcome after transplantation associated with travel time by assessing regraft‐free survival between groups of travel time. Of 8490 patients transplanted, 3050 required retransplantation or died within median follow‐up of 1596 days (IQR 536‐3306 days). The results of univariable analyses between travel time categories are presented in Tables 1 and 2, and the results were consistent with those for the cohort of listed patients, as previously described. An unadjusted analysis of survival demonstrated no significant differences between travel‐time categories (*P = *.645 by log‐rank test) (Figure S8). A multivariable Cox proportional hazards model also found no significant difference in HR according to travel time category, after accounting for other potentially confounding factors (*P = *.532, Table 4), with a HR of 0.99 (95% CI: 0.82‐1.19, *P = *.886) for the >119‐minute group vs <60‐minute group. Consistent results were returned from sensitivity analyses where assessments for distance from transplant center or with travel time as a continuous variable were considered (data not shown).

**Table 4:** Cox model results for retransplant‐free survival following liver transplantation

Variable	Hazard ratio (95% CI)	*P*
Travel time tertile		.532
<60 min	1.00	—
60‐119 min	0.91 (0.79‐1.05)	.207
>119 min	0.99 (0.82‐1.19)	.886
Primary liver disease		**<.001**
PBC	1.00	—
AIH	1.34 (0.93‐1.94)	.120
HBV	1.02 (0.68‐1.55)	.910
PSC	1.96 (1.51‐2.55)	**<.001**
Other	1.39 (1.07‐1.81)	**.014**
Alcohol	1.29 (1.01‐1.65)	**.040**
HCV	1.75 (1.37‐2.24)	**<.001**
NAFLD	1.66 (1.16‐2.36)	**.006**
CMV status		**.015**
Donor− Recipient−	1.00	—
Donor− Recipient+	0.99 (0.83‐1.19)	.936
Donor+ Recipient+	1.21 (1.01‐1.45)	**.040**
Donor+ Recipient−	0.98 (0.80‐1.20)	.842
Serum creatinine (/100 µmol/L)	1.20 (1.03‐1.40)	**.021**
INR (per 2‐fold increase)	0.58 (0.42‐0.81)	**.001**
Donor age (/10 y)	1.08 (1.03‐1.13)	**<.001**
Transplant center		**.001**
King's College	1.00	—
Leeds	1.56 (1.15‐2.10)	**.004**
Birmingham	1.46 (1.13‐1.89)	**.004**
Edinburgh	1.64 (1.23‐2.19)	**.001**
Cambridge	1.73 (1.31‐2.30)	**<.001**
Royal Free	1.40 (1.05‐1.87)	**.021**
Newcastle	1.52 (1.08‐2.12)	**.015**
Cold ischemic time (/100 min)	1.06 (1.01‐1.10)	**.011**
Days in ITU (per doubling in d)	1.35 (1.28‐1.44)	**<.001**
Listing year (/10 y)	0.68 (0.54‐0.86)	**.001**
Recipient ethnicity		.052
White	1.00	—
Asian	0.91 (0.72‐1.16)	.458
Black	1.38 (0.94‐2.02)	.100
Other/mixed	0.19 (0.05‐0.77)	**.020**
Transplant type		
DBD	1.00	**—**
DCD	1.66 (1.38‐1.99)	**<.001**

AIH, autoimmune hepatitis; BMI, body mass index; CI, confidence interval; CMV, cytomegalovirus; DBD, donation after brain death; DCD, donation after cardiac death; HBV, hepatitis B virus; HCC, hepatocellular carcinoma; HCV, hepatitis C virus; INR, international normalized ratio; IQR, interquartile range; ITU, intensive treatment unit; NAFLD, nonalcoholic fatty liver disease; PBC, primary biliary cholangitis; PSC, primary sclerosing cholangitis.

Variables presented represent those from Tables 1 and 2 retained in the competing risks model after backwards stepwise selection: covariables excluded because of the absence of a significant effect on outcome were: Age, sex, BMI, serum bilirubin, serum sodium, INR, diabetes mellitus, hemodialysis, HCC, donor age, cold ischemic time, days in ITU, transplant weekday, CMV status, donor sex, and donor ethnicity. Hazard ratios (HR) are presented along with upper and lower 95% confidence intervals. Only listings with complete data are included (n = 4158). The HR calculated for INR and days in ITU relates to an increase of 1 in the log_2_‐transformed variable, eg, a 2‐fold increase in INR. For the other continuous factors, HRs are for increases of the stated number of units. Blood results are from the point of listing. Bold *P *values are significant at *P* < .05.

### 3.4 The optimum site for an additional UK transplant center to reduce patient travel time is Bristol

**Figure 6:**
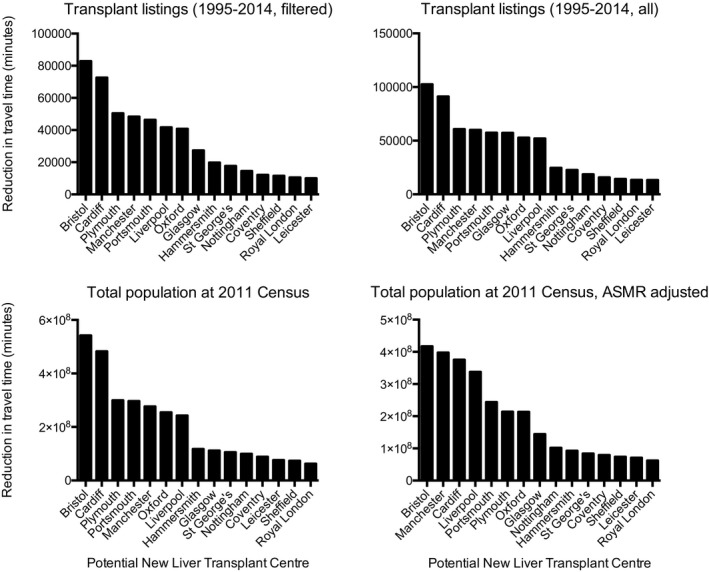
Modeled reductions in travel time by introduction of a new liver transplant center at various locations. The models described elsewhere in this document were repeated with the addition of a hypothetical new liver transplant center at each of 15 locations. The reduction in the number of minutes of traveling time for each listing for transplantation, or member of the population, to attend their nearest liver transplant center with and without the modeled new center was calculated. Panel A represents all listings for liver transplantation 1995‐2014 after the application of our exclusion criteria; Panel B represents all listings without exclusions; Panel C represents travel for the population of England, Scotland, and Wales as at the 2011 National Census; Panel D represents the same Census population but normalized to age‐adjusted standardized mortality rates (ASMR)

Having demonstrated a correlation between increasing travel time and worsened outcome, we modeled the effect on total travel time to the nearest liver transplant center of an additional liver center. An additional center was modeled at each of the existing UK mainland renal transplant centers without current liver transplant facilities: Bristol, Cardiff, Coventry, Glasgow, Leicester, Liverpool, St George’s Hospital (London), the West London Renal and Transplant Centre (“Hammersmith,” London), The Royal London Hospital, Manchester, Nottingham, Oxford, Plymouth, Portsmouth, and Sheffield (15 centers; Figure S9A). We calculated the reduction in person minutes of travel time that the introduction of a new center would produce in each of 4 scenarios: for all liver transplant patients listed 1995‐2014 after the exclusions described above (Figure 6A), for all listed patients without exclusions applied (Figure 6B), for the total population (Figure 6C), and for the total population with adjustment for ASMR (Figure 6D). In each case, the greatest overall reduction in patient travel time was achieved by modeling the addition of a new center in Bristol. For liver transplant listings after exclusions, a predicted saving of 82 913 minutes (10.1%) was predicted with a center in Bristol, with other centers ranging from 72 603 minutes (8.8%) with Cardiff to 10 063 minutes with Leicester (1.2%); analyses using different inclusion criteria returned consistent results. A map showing the effects of a new center at Bristol is shown as Figure S9B.

## 4 DISCUSSION

Here we show that there is a significant disparity in travel time to liver transplant services across the United Kingdom. For those listed for transplantation, greater travel time to transplant center correlates with a worse outcome: a greater likelihood of death while listed and a lower likelihood of receiving a transplant. However, in contrast to reports from the United States, there was no apparent difference in outcome after transplantation for those living further from liver transplant centers.

We show that approximately 7.5 million people (around 12.5% of the population) live >2 hours travel from a liver transplant center in the United Kingdom. There are variations in disease pathogenesis with geography, with fewer people distant from a transplant center being listed for both hepatitis B‐ and C‐related disease, perhaps reflecting the urban concentration of hepatitis and associations with ethnic grouping and relative wealth. Patients are also traveling further with time. A number of other factors including both recipient and donor ethnicity, donor sex blood groups, cytomegalovirus status, serum bilirubin, time spent in intensive treatment unit, and cold ischemic time varied significantly by distance. The first 4 of these might reasonably be explained by variations in the populations using the individual transplant centers, while the causes of variations in the last 3 are less apparent. A trend to higher bilirubin in those traveling further may be consistent with delays in referral for transplantation,^20^ but this study lacks details of the denominator population that would be required to fully explore this. Interestingly, cold ischemic time was lower for those living further away from transplant centers. One possible explanation for this finding is patients living nearby being called in rapidly as second‐choice candidate after a problem with the first choice.

Our findings of a significant correlation between travel time to transplant center and worsened outcome after listing are consistent with the findings of 2 large US studies,^5^, ^6^ but not all analyses of the US system.^20^, ^21^ This study is not designed to explain why such variations in outcome may occur, but potential explanations include less frequent pretransplant follow‐up or less specialized pretransplant care, delays in referral for transplantation not reflected in the covariables that we have, or an unmeasured preference for offering organs to those geographically closer. Such differences warrant further examination and provide impetus to efforts to reduce such disparities in access to liver transplantation. In addition, we cannot account for possible regional variations in the approach to listing or delisting patients. Indeed, differences in the general behavior of clinicians and/or patients further from transplant centers may also explain some of the variation in outcome we describe. Such variation might be amenable to educational approaches. However, major differences in behavior might also be expected to have effects on posttransplant outcomes, and these were not evident in this study.

In contrast to work from the United States, we do not, however, show variations in outcome after transplantation in those living further from their transplanting center. One possible reason is that the geography of the United Kingdom means that the most distant patients are still nearer a transplant center than their counterparts in the United States. However, in the analysis by Goldberg et al, differences in outcome were apparent when comparing 0‐100 and 100‐200 miles of distance. Such distances are within those seen in the United Kingdom. In addition, the correlation of rurality or socioeconomic status and travel time from liver transplant center may differ between the United States and the United Kingdom and are not addressed in our study.

A further consideration is the different organization of the UK and US healthcare systems: The NHS represents a single system with a semiformalized referral network,^22^ whereas multiple providers and funders contribute to the United Network for Organ Sharing network. Similar analyses of the effect of travel time and distance are not available for other liver transplant systems—either centralized or more diffuse.

Having demonstrated a correlation between greater travel time to transplant center and worsened outcome, we continued to model where a new liver transplant center might optimally be placed. We also note that patient preference, the logistics of organ procurement, and potential benefits to referral networks and pre/posttransplant care are also potential reasons for wishing to minimize distance between patients and transplant center, but are not examined here. Our finding of the optimal site for a new liver transplant center differs from that expected by a simple inspection of distribution of the frequency of severe liver disease as measured by ASMR: In the United Kingdom, mortality rates from liver disease are greatest in the northwest of England. The finding that ≈25% of patients listed for liver transplantation are seen in a center that is not their geographically closest suggests that factors other than travel time are also important: possibilities include physician referral patterns,^22^ change of residence after initial referral, and differences in specialties between centers including for rarer causes.

Although our data set is relatively large with excess of 11 000 listings considered, one potential concern is that with the multiple variables examined, an otherwise statistically significant outcome signal from travel time posttransplantation might be lost. This is made less likely by significant differences in HRs for posttransplant outcome in risk factors in other studies (eg, increased mortality in those receiving organs from deceased after cardiac death donors, those receiving grafts from older donors or with longer cold ischemic times, and those with renal failure at listing).^23^, ^24^

Weaknesses of this analysis include the moderate imprecision introduced by only using the first part of the postal code, although this was necessary to preserve relative anonymity, and these factors represent potential confounders of our findings. This imprecision also precludes estimates of social status and income based on place of residence, although we note that population rates of mortality from liver disease tend to be lower with greater travel time. Importantly, we only considered those who reached the point of listing for transplantation and are therefore unable to account for geographic variations in ability to access assessment for possible liver transplantation. We have, however, attempted to account for this in our geographic analysis by using ASMR as a proxy for total liver disease. It is notable, however, that a large proportion of liver‐related death in the United Kingdom is alcohol related and that these patients are often not referred for consideration of transplantation.^12^ In addition, we only considered mortality and a requirement for retransplantation as outcomes; it is possible that patient experience, loss of productive work, financial cost, and other variables are affected by distance from transplant center. We were also unable to ascertain which patients changed their address; patient migration for the purposes of transplantation is reportedly common in the United States.^25^ We recorded variation in outcome between centers in the United Kingdom both from the point of listing and from the point of transplantation. However, for the reasons explored above and because of the lack of information about the denominator population including those who are not accepted for listing, further work would be necessary to understand this variation. Finally, in our modeling we do not consider the addition of more than one center to the current network.

The issue of how best to approach the provision of liver transplantation in the United Kingdom is challenging. We highlight disparity in access to liver transplant centers and demonstrate a correlation between greater distance from transplant center and outcome from the point of listing for transplantation, although we do not prove causation. Further careful analysis will be required to guide future decisions on both the number and geographical distribution of liver transplant centers, including the consideration of factors other than simple mortality and chance of attaining transplantation.

## DISCLOSURE

The authors of this manuscript have no conflicts of interest to disclose as described by the *American Journal of Transplantation*.

## CONTRIBUTIONS

GJW originally conceived the study, which was then modified in response to suggestions from each of the other authors. The analysis was performed by GJW and JH. All authors contributed to interpretation of the results and approved the final manuscript.

## DISCLAIMER

This report presents independent research funded by the National Institute for Health Research (NIHR). The views expressed are those of the authors(s) and not necessarily those of the NHS, the NIHR, or the Department of Health.

## References

[1] Zarrinpar A , Busuttil RW . Liver transplantation: past, present and future. Nat Rev Gastroenterol Hepatol. 2013;10(7):434‐440. 23752825 10.1038/nrgastro.2013.88

[2] Mokdad AA , Lopez AD, Shahraz S, et al. Liver cirrhosis mortality in 187 countries between 1980 and 2010: a systematic analysis. BMC Med. 2014;12:145. 25242656 10.1186/s12916-014-0145-y PMC4169640

[3] Moylan CA , Brady CW, Johnson JL, Smith AD, Tuttle‐Newhall JE, Muir AJ. Disparities in liver transplantation before and after introduction of the MELD score. JAMA. 2008;300(20):2371‐2378. 19033587 10.1001/jama.2008.720 PMC3640479

[4] Gentry SE , Massie AB, Cheek SW, et al. Addressing geographic disparities in liver transplantation through redistricting. Am J Transplant. 2013;13(8):2052‐2058. 23837931 10.1111/ajt.12301 PMC4674218

[5] Zorzi D , Rastellini C, Freeman DH, Elias G, Duchini A, Cicalese L. Increase in mortality rate of liver transplant candidates residing in specific geographic areas: analysis of UNOS data. Am J Transplant. 2012;12(8):2188‐2197. 22845911 10.1111/j.1600-6143.2012.04083.x PMC3410658

[6] Goldberg DS , French B, Ford KA, et al. Association of distance from a transplant center with access to waitlist placement, receipt of liver transplantation, and survival among US veterans. JAMA. 2014;311(12):1234. 24668105 10.1001/jama.2014.2520 PMC4586113

[7] Axelrod DA , Dzebisashvili N, Schnitzler MA, et al. The interplay of socioeconomic status, distance to center, and interdonor service area travel on kidney transplant access and outcomes. Clin J Am Soc Nephrol. 2010;5(12):2276‐2288. 20798250 10.2215/CJN.04940610 PMC2994090

[8] Ambroggi M , Biasini C, Del Giovane C, Fornari F, Cavanna L. Distance as a barrier to cancer diagnosis and treatment: review of the literature. Oncologist. 2015;20(12):1378‐1385. 26512045 10.1634/theoncologist.2015-0110 PMC4679078

[9] Gooiker GA , Lemmens V, Besselink MG, et al. Impact of centralization of pancreatic cancer surgery on resection rates and survival. Br J Surg. 2014;101(8):1000‐1005. 24844590 10.1002/bjs.9468

[10] Edwards EB , Roberts JP, McBride MA, Schulak JA, Hunsicker LG. The effect of the volume of procedures at transplantation centers on mortality after liver transplantation. N Engl J Med. 1999;341(27):2049‐2053. 10615076 10.1056/NEJM199912303412703

[11] British Association for the Study of the Liver and British Society of Gastroenterology Liver Section. A time to act: improving liver health and outcomes in liver disease. The National Plan for Liver Services. 2009.

[12] Williams R , Aspinall R, Bellis M, et al. Addressing liver disease in the UK: a blueprint for attaining excellence in health care and reducing premature mortality from lifestyle issues of excess consumption of alcohol, obesity, and viral hepatitis. Lancet. 2014;384(9958):1953‐1997. 25433429 10.1016/S0140-6736(14)61838-9

[13] Williams R , Alexander G, Aspinall R, et al. New metrics for the lancet standing commission on liver disease in the UK. Lancet. 2017;389:2053‐2080. 27989558 10.1016/S0140-6736(16)32234-6

[14] Williams R , Alexander G, Armstrong I, et al. Disease burden and costs from excess alcohol consumption, obesity, and viral hepatitis: fourth report of the Lancet Standing Commission on Liver Disease in the UK. Lancet. 2018;391:1097‐1107. 29198562 10.1016/S0140-6736(17)32866-0

[15] Julie Ratcliffe MB . Patient's preferences regarding the process and outcomes of life‐saving technology. Int J Technol Assess Healthcare. 1999;15(2):340‐351. 10507193

[16] Cuzick J . A Wilcoxon‐type test for trend. Stat Med. 1985;4(1):87‐90. 3992076 10.1002/sim.4780040112

[17] Spiegelhalter DJ . Funnel plots for comparing institutional performance. Stat Med. 2005;24(8):1185‐1202. 15568194 10.1002/sim.1970

[18] Fine JP , Gray RJ . A proportional hazards model for the subdistribution of a competing risk. J Am Stat Assoc. 1999;94(446):496‐509.

[19] Zhou B , Fine J , Laird G . Goodness‐of‐fit test for proportional subdistribution hazards model. Stat Med. 2013;32(22):3804‐3811. 23625840 10.1002/sim.5815

[20] Axelrod DA , Guidinger MK , Finlayson S, et al. Rates of solid‐organ wait‐listing, transplantation, and survival among residents of rural and urban areas. JAMA. 2008;299(2):202‐207. 18182602 10.1001/jama.2007.50

[21] Firozvi AA , Lee CH , Hayashi PH . Greater travel time to a liver transplant center does not adversely affect clinical outcomes. Liver Transpl. 2008;14(1):18‐24. 18161800 10.1002/lt.21279

[22] O'Grady JG . Network and satellite arrangements in liver disease. Frontline Gastroenterol. 2013;4(3):187‐190. 23795284 10.1136/flgastro-2012-100267 PMC3686327

[23] Callaghan CJ , Charman SC , Muiesan P , et al. Outcomes of transplantation of livers from donation after circulatory death donors in the UK: a cohort study. BMJ Open. 2013;3(9):e003287. 10.1136/bmjopen-2013-003287 PMC3773642 24002984

[24] Adam R , Karam V, Delvart V, et al. Evolution of indications and results of liver transplantation in Europe. A report from the European Liver Transplant Registry (ELTR). J Hepatol. 2012;57(3):675‐688. 22609307 10.1016/j.jhep.2012.04.015

[25] Croome KP , Lee DD , Burns JM , Perry DK , Keaveny AP , Taner CB . Patterns and outcomes associated with patient migration for liver transplantation in the United States. PLoS One. 2015;10(10):e0140295. 26469071 10.1371/journal.pone.0140295 PMC4607372

