# Supplementary figures and images for: Proximity to transplant center and outcome among liver transplant patients
Source: Am J Transplant. 2018 Aug 3;19(1):208–20. doi: 10.1111/ajt.15004 (PMC6491997; doi:10.1111/ajt.15004)

Figure S1

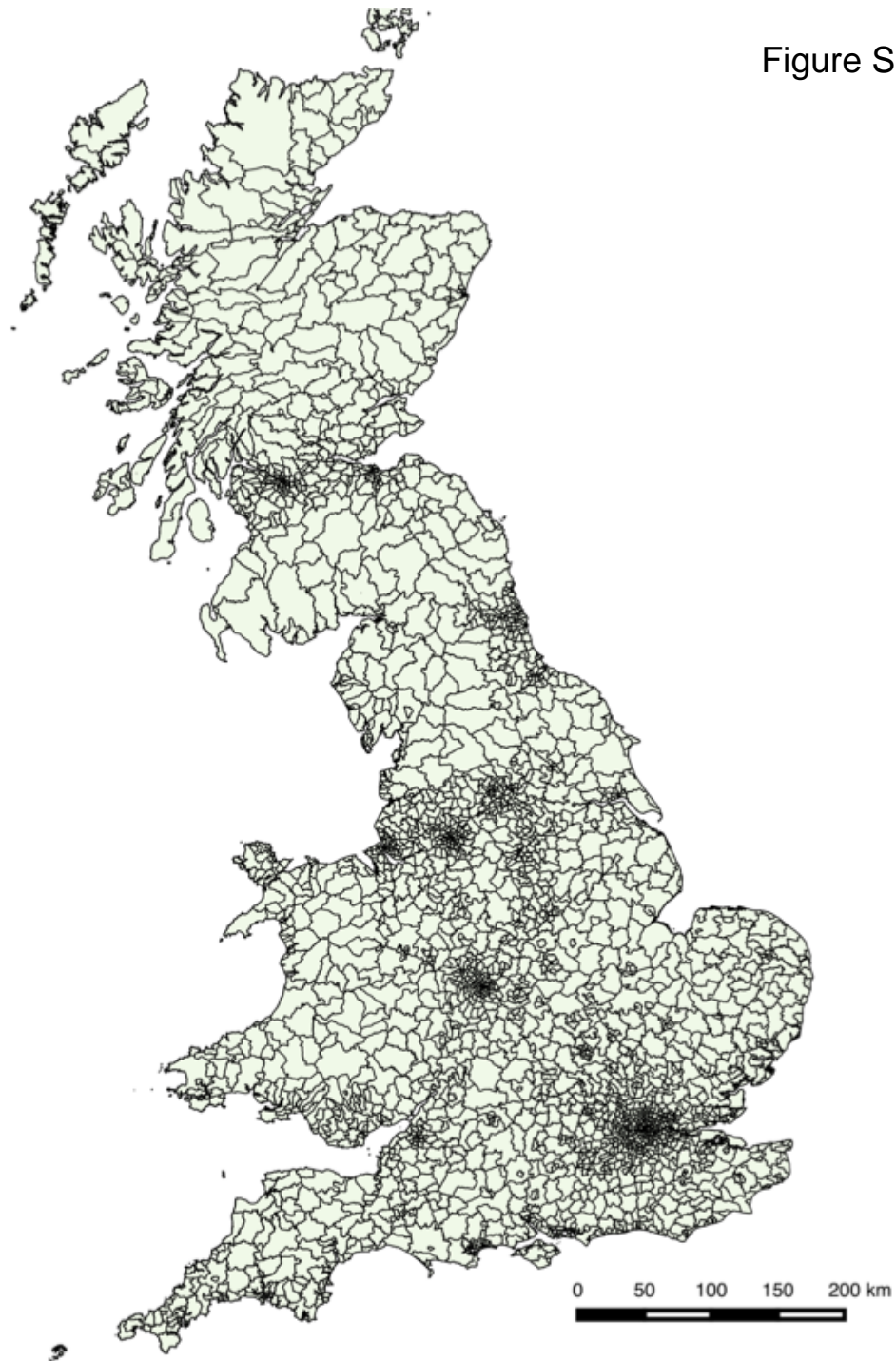

Supplement: Supplementary file 1 [file AJT-19-208-s001.pdf]

Figure S2

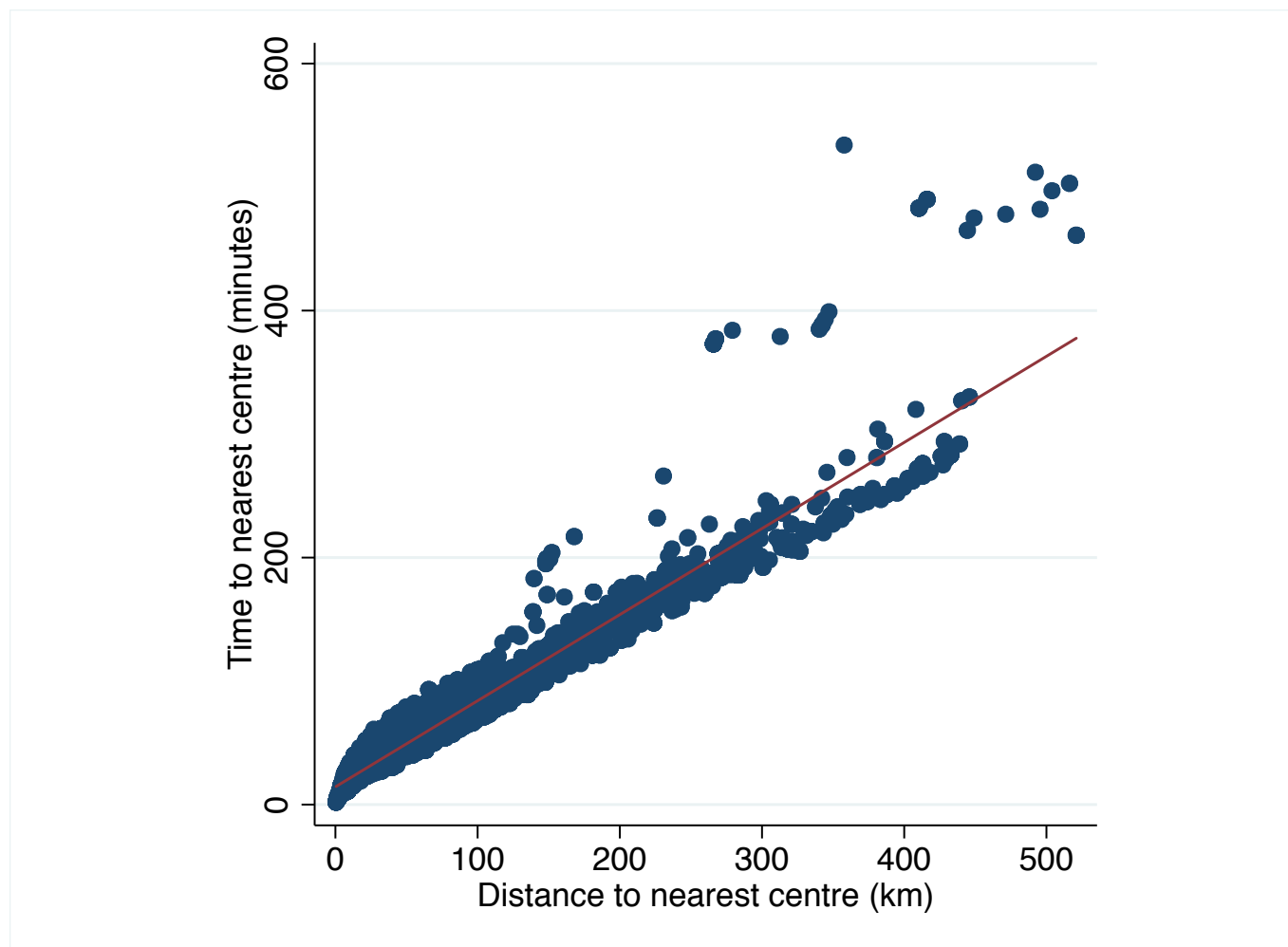

Supplement: Supplementary file 2 [file AJT-19-208-s002.pdf]

Figure S3

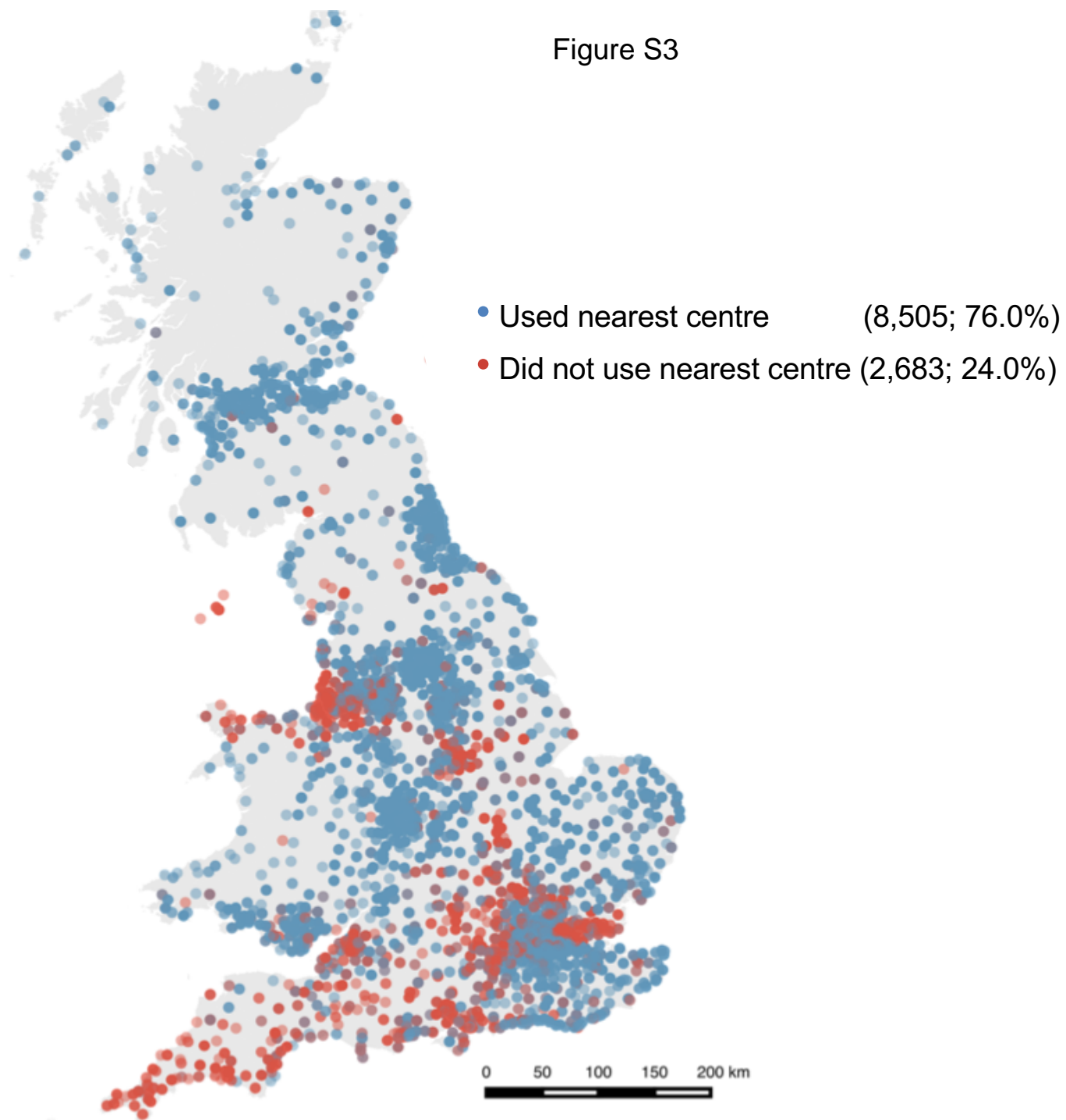

Supplement: Supplementary file 3 [file AJT-19-208-s003.pdf]

Figure S4

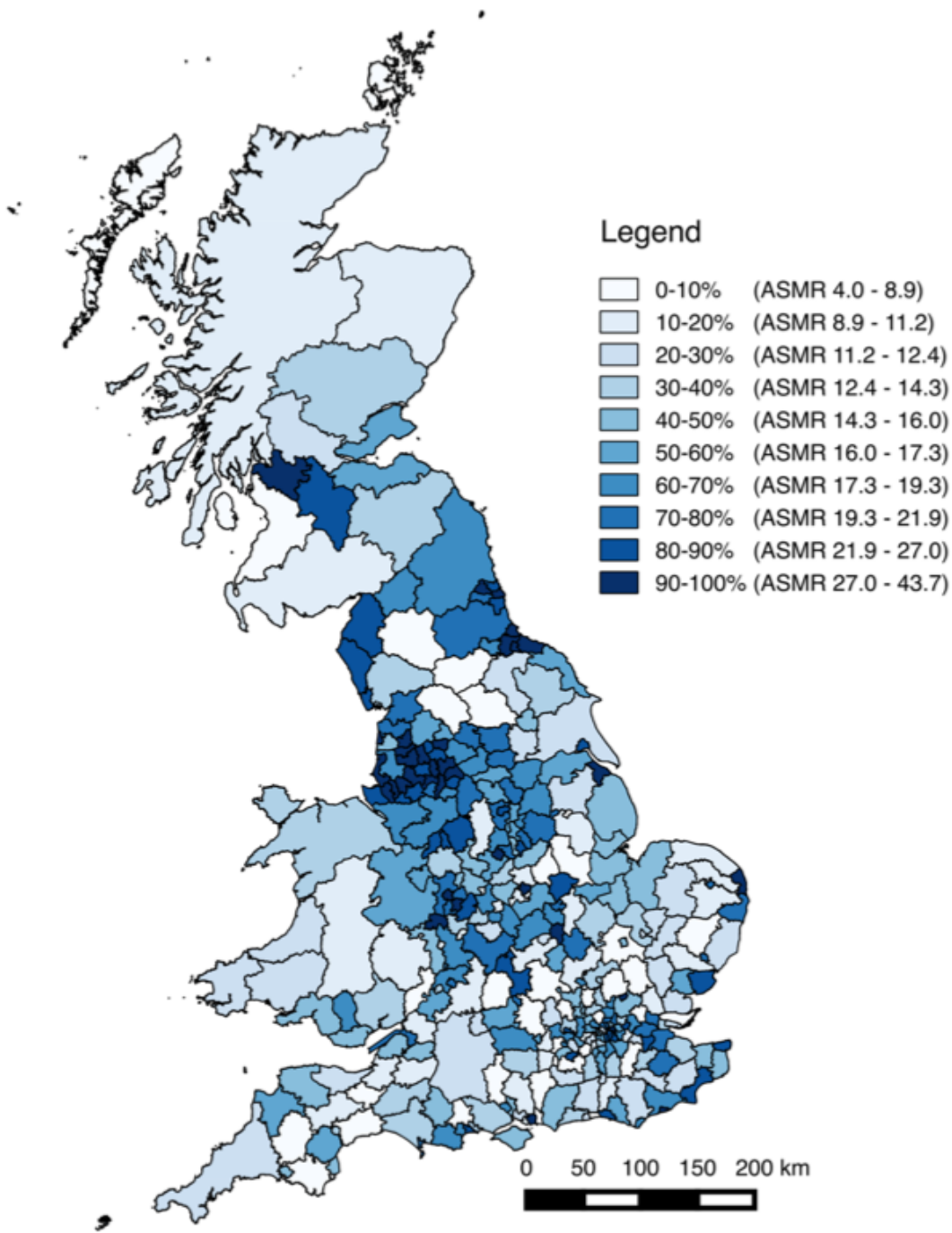

Supplement: Supplementary file 4 [file AJT-19-208-s004.pdf]

Figure S5

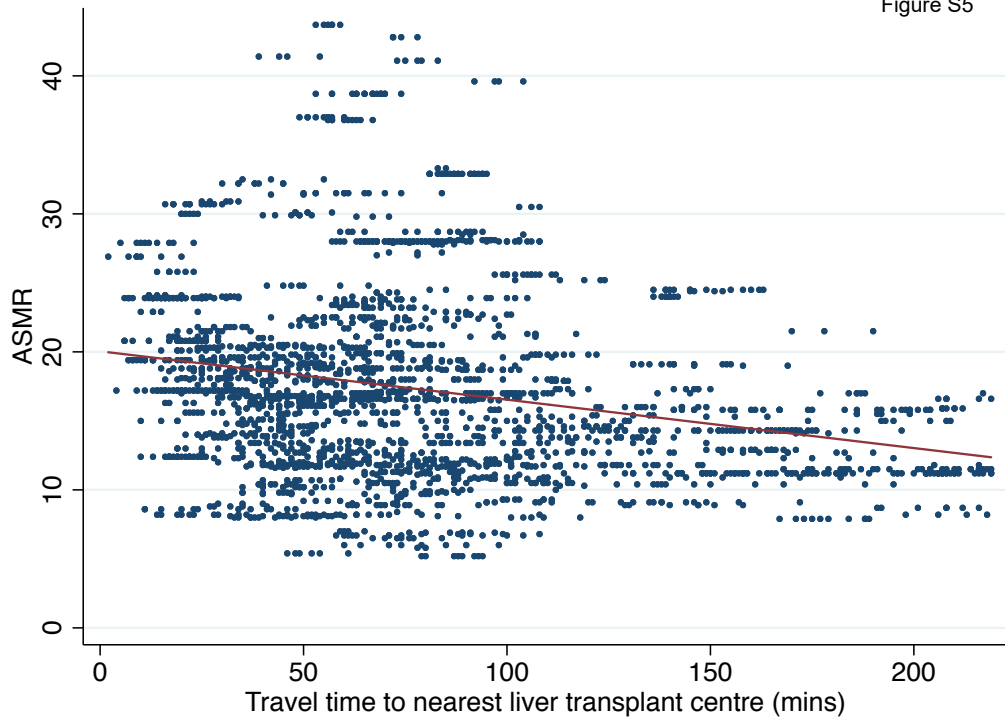

Supplement: Supplementary file 5 [file AJT-19-208-s005.pdf]

Figure S6

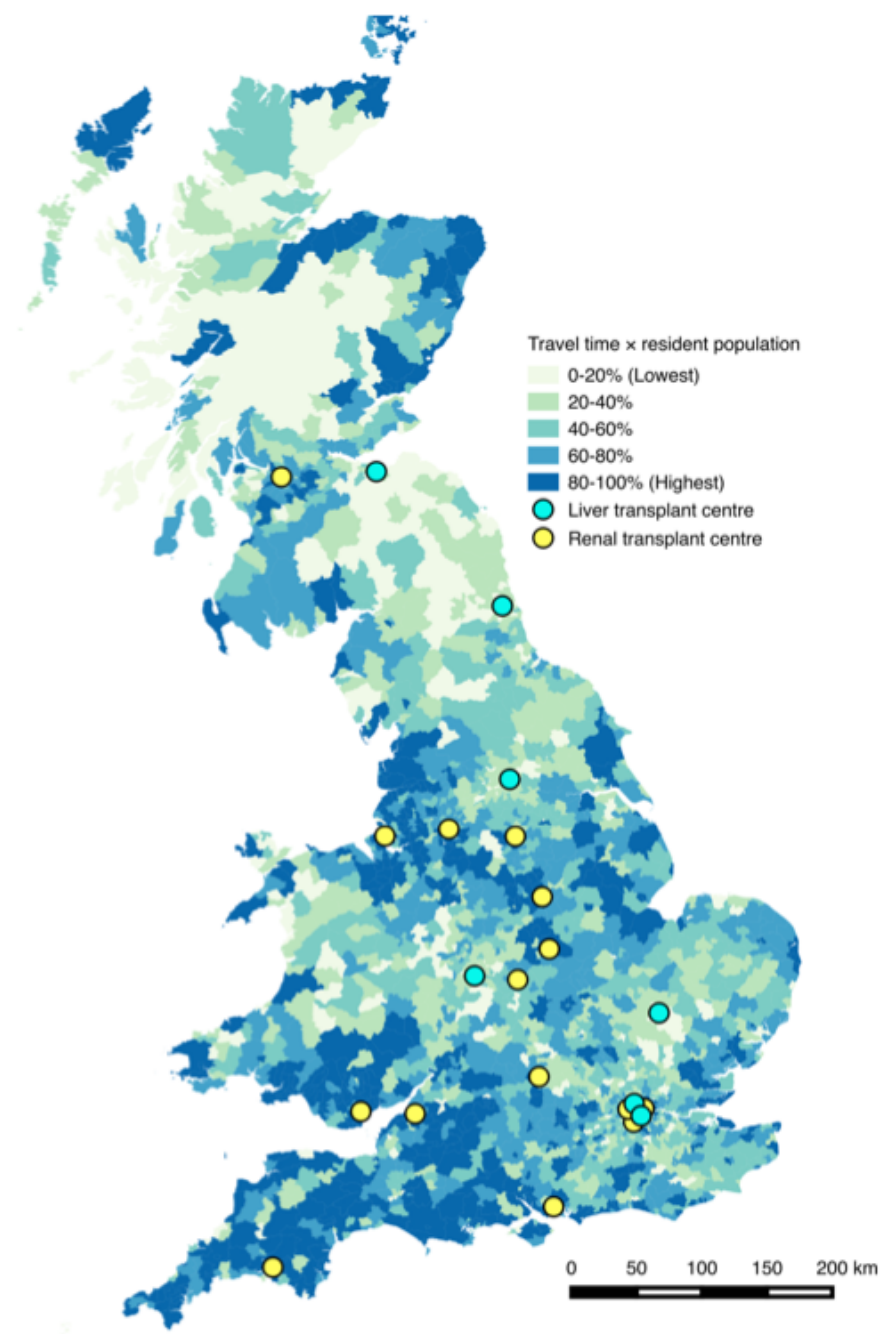

Supplement: Supplementary file 6 [file AJT-19-208-s006.pdf]

Figure S7

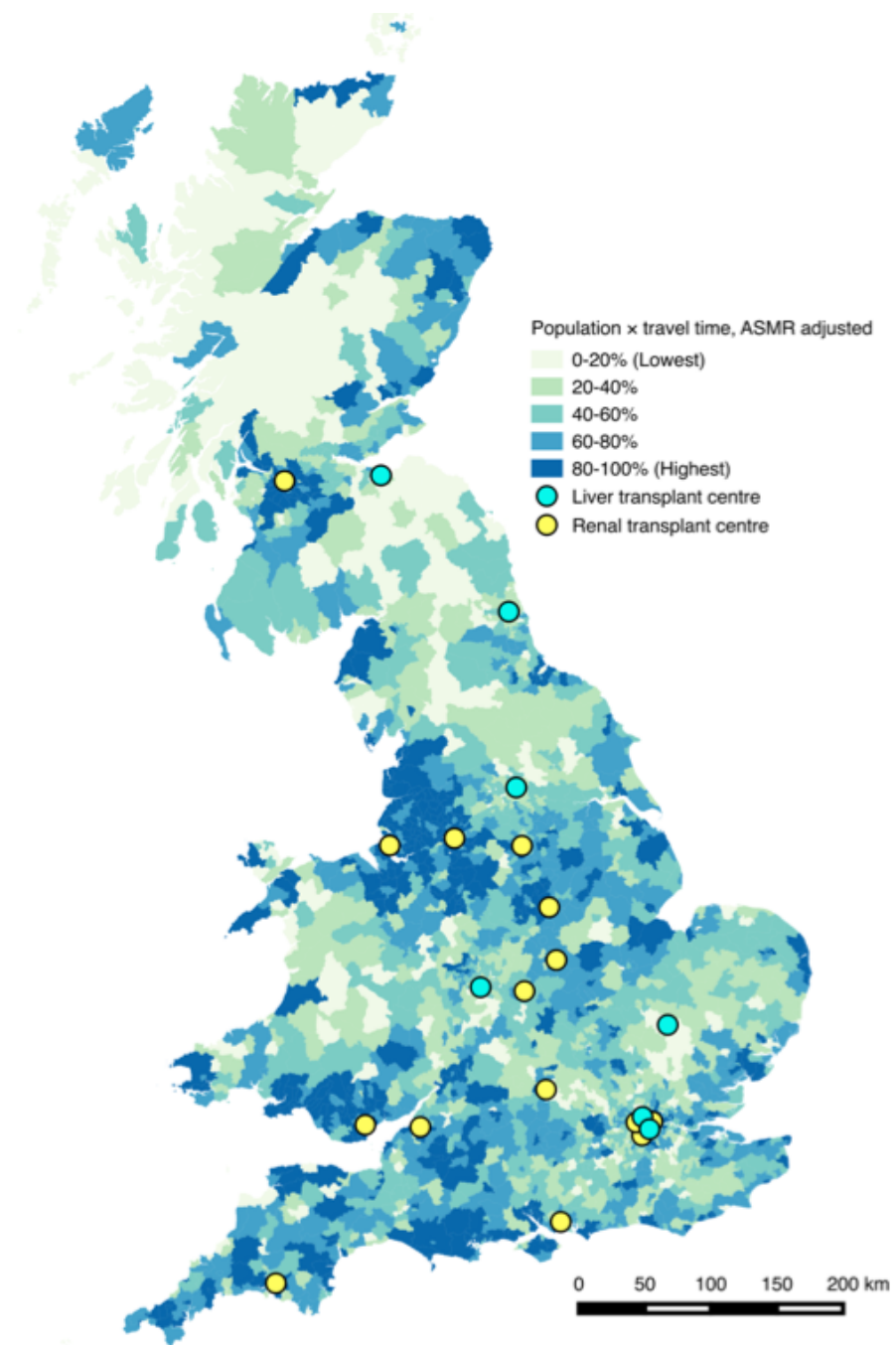

Supplement: Supplementary file 7 [file AJT-19-208-s007.pdf]

Figure S8

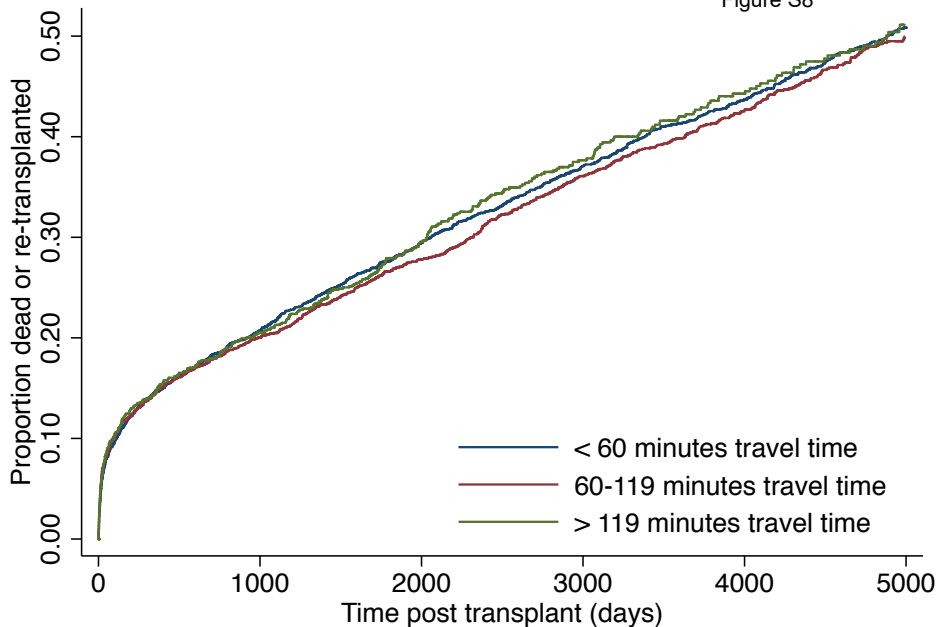

Number at risk

|                |      |      |      |      |     |     |
|----------------|------|------|------|------|-----|-----|
| <60 minutes    | 4003 | 2557 | 1757 | 1171 | 800 | 472 |
| 60-119 minutes | 3133 | 1964 | 1363 | 903  | 600 | 326 |
| > 119 minutes  | 1336 | 840  | 560  | 358  | 221 | 132 |

Supplement: Supplementary file 8 [file AJT-19-208-s008.pdf]

Figure S9

A

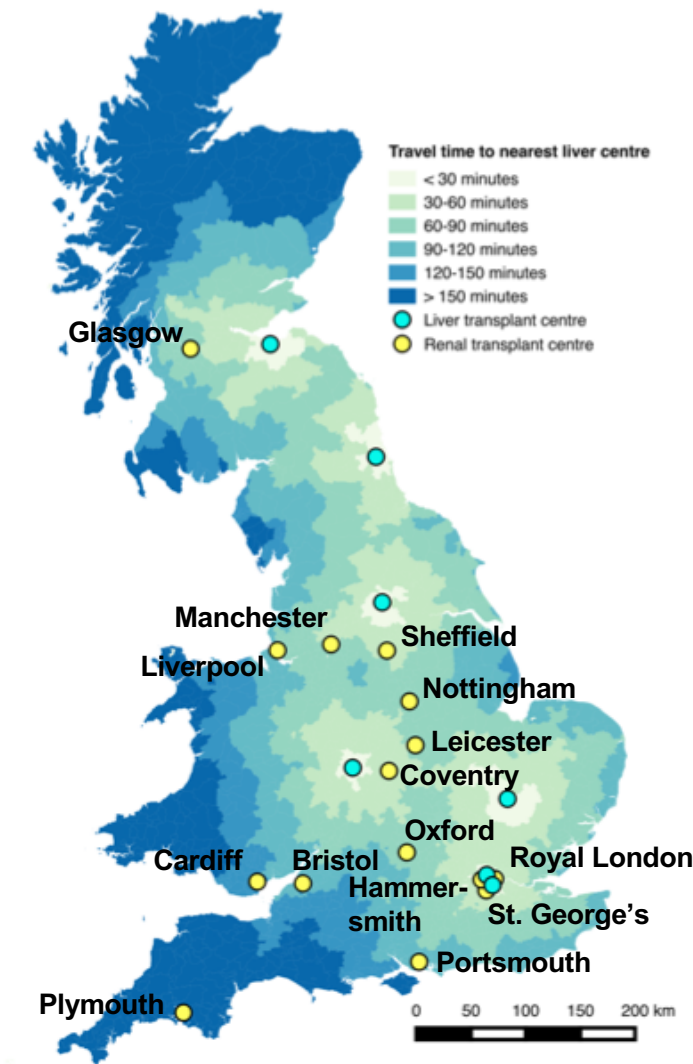

B

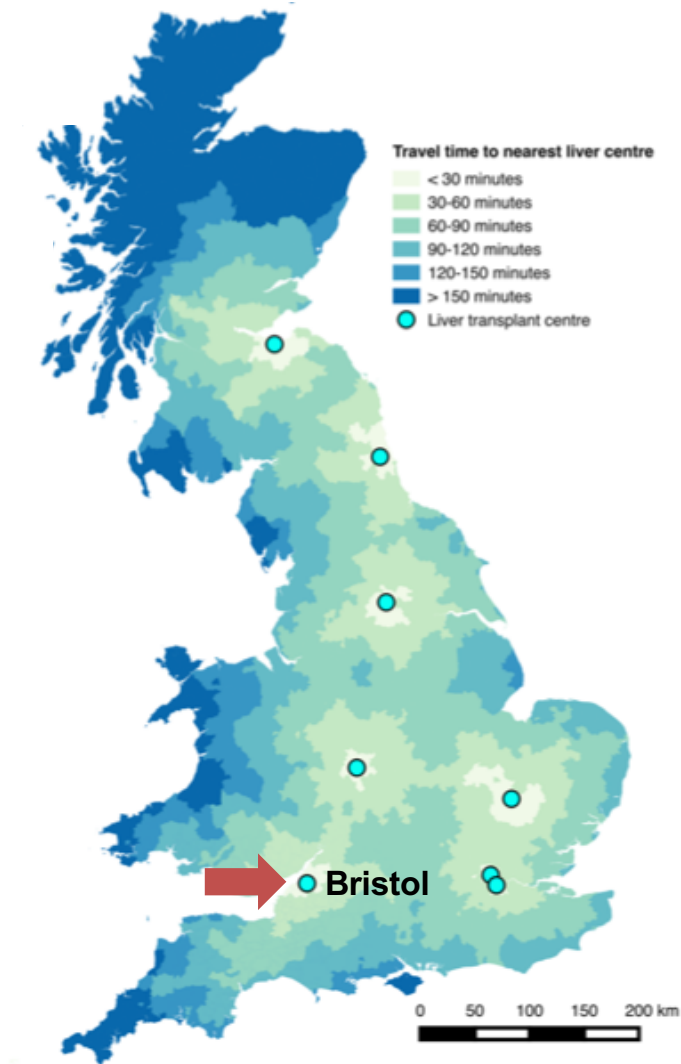

Supplement: Supplementary file 9 [file AJT-19-208-s009.pdf]
