# Supplementary material for: Proximity to transplant center and outcome among liver transplant patients
Source: Am J Transplant. 2018 Aug 3;19(1):208–20. doi: 10.1111/ajt.15004 (PMC6491997; doi:10.1111/ajt.15004)
Supplement: Supplementary file 10 [file AJT-19-208-s010.docx]

# Supplementary tables

### Supplementary table 1 – Data sources used

| **Data** | **Source** |
| --- | --- |
| Core dataset of liver listings and transplants | NHS Blood and Transplant |
| Age-adjusted standardised mortality ratios | http://data.gov.uk  http://healthmapswales.nhs.uk  http://scotpho.org.uk |
| Postcode centroids | https://geoportal.statistics.gov.uk/ |
| NHS Healthcare Administrative subunit boundaries | https://www.england.nhs.uk/resources/ccg-maps/  http://www.sharegeo.ac.uk/handle/10672/333  https://data.gov.uk/dataset/nhs-health-boards |
| National population data | https://www.ons.gov.uk/census/2011census |
| Google Maps API | https://developers.google.com/maps/ |
| Maps of the United Kingdom and postcode boundaries | Ordnance Survey Opendata: http://www.ordnancesurvey.co.uk |
| Details of liver and renal transplant centres | www.odt.nhs.uk/transplantation/transplant-units-in-uk/ |

### Supplementary Table 2 – Likelihood of transplantation or recovery from the point of listing for liver transplantation

### Table footnote: Competing risks analysis with transplantation or recovery and death or deterioration otherwise preventing transplantation as alternative outcomes. Variables presented represent those from Table 1 and 2 retained in the competing risks model after backwards selection. Subdistribution hazard ratios (sHR) are presented along with upper and lower 95% confidence intervals. Only listings with complete data are included (n = 6,744). The HR calculated for INR and days in ITU relates to an increase of one in the log_2_-transformed variable, e.g. a two-fold increase in INR. Blood results are from the point of listing. Bold p-values are significant at p<0.05. AIH = autoimmune hepatitis; BMI = body mass index; CI = confidence interval; HBV = hepatitis B virus; HCC = hepatocellular carcinoma; HCV = hepatitis C virus; INR = international normalised ratio; IQR = interquartile range; NAFLD = non-alcoholic fatty liver disease; PBC = primary biliary cholangitis; PSC = primary sclerosing cholangitis.

| **Variable** | **sHR (95%CI)** | **p** |
| --- | --- | --- |
| **Travel time tertiles:** | - | **<0.001** |
| < 60 mins | 1.00 | - |
| 60-119 mins | 0.94 (0.88-0.99) | **0.039** |
| > 119 mins | 0.86 (0.79-0.93) | **<0.001** |
| **Age (/10 years)** | 0.94 (0.91-0.96) | **<0.001** |
| **Serum creatinine (/100µmol/L)** | 0.73 (0.67-0.80) | **<0.001** |
| **INR (per twofold increase)** | 1.24 (1.06-1.45) | **0.006** |
| **Sex:** | - | - |
| Male | 1.00 | - |
| Female | 0.88 (0.83-0.94) | **<0.001** |
| **Transplant centre:** | - | **-** |
| King's College | 1.00 | - |
| Leeds | 0.95 (0.87-1.05) | 0.330 |
| Birmingham | 1.31 (1.20-1.43) | **<0.001** |
| Edinburgh | 1.25 (1.13-1.39) | **<0.001** |
| Cambridge | 1.23 (1.11-1.36) | **<0.001** |
| Royal Free | 0.96 (0.86-1.06) | 0.401 |
| Newcastle | 1.07 (0.94-1.21) | 0.290 |
| **HCC:** | - | **-** |
| No | 1.00 | - |
| Yes | 1.38 (1.27-1.5) | <0.001 |
| **Blood group:** | - | **-** |
| O | 1.00 | - |
| A | 1.65 (0.55-1.76) | **<0.001** |
| B | 1.08 (0.98-1.17) | 0.108 |
| AB | 2.05 (1.74-2.41) | **<0.001** |
| **Primary liver disease:** | - | **<0.001** |
| PBC | 1.00 | - |
| AIH | 0.88 (0.75-1.03) | 0.112 |
| HBV | 1.09 (0.91-1.29) | 0.363 |
| PSC | 1.03 (0.91-1.16) | 0.682 |
| Other | 0.66 (0.58-0.74) | **<0.001** |
| Alcohol | 0.89 (0.80-1.00) | **0.045** |
| HCV | 0.87 (0.77-0.97) | **0.017** |
| NAFLD | 0.78 (0.67-0.91) | **0.002** |
| **Listing year (/10 years)** | 0.76 (0.69-0.84) | **<0.001** |
